# Plasma levels of Galectin-9 reflect disease severity in malaria infection

**DOI:** 10.1186/s12936-016-1471-7

**Published:** 2016-08-11

**Authors:** Bindongo P. P. Dembele, Haorile Chagan-Yasutan, Toshiro Niki, Yugo Ashino, Noppadon Tangpukdee, Egawa Shinichi, Srivicha Krudsood, Shigeyuki Kano, Toshio Hattori

**Affiliations:** 1 Division of International Cooperation for Disaster Medicine, International Research Institute of Disaster Science, Tohoku University, Sendai, Japan; 2 Division of Disaster-related Infectious Disease, International Research Institute of Disaster Science, Tohoku University, Sendai, Japan; 3 Emerging Infectious Diseases, Graduate School of Medicine, Tohoku University, Sendai, Japan; 4 Department of Immunology, Kagawa University, Takamatsu, Japan; 5 GalPharma Co., Ltd., Takamatsu, Japan; 6 Department of Clinical Tropical Medicine, Faculty of Tropical Medicine, Mahidol University, Bangkok, Thailand; 7 Clinical Malaria Research Unit, Faculty of Tropical Medicine, Mahidol University, Bangkok, Thailand; 8 Department of Tropical Medicine and Malaria, Research Institute, National Center for Global Health and Medicine, Tokyo, Japan; 9 Department of Occupational Therapy, Graduate School of Health Science Studies, Kibi International University, 8 Igamachi, Takahashi, Okayama Japan

## Abstract

**Background:**

Galectin-9 (Gal-9) is a β-galactoside-binding lectin that interacts with sugar moieties on glycoproteins and glycolipids of cells and pathogens. Gal-9 is known as an immune modulator that induces cell death via interaction with T cell immunoglobulin and mucin domain-3 (Tim3), a co-inhibitory receptor, and it inhibits production of several pro-inflammatory cytokines (TNF, IL-6 and IL-1α) and enhances production of IL-10. To understand the immune pathology of malaria, the Gal-9 in plasma was measured.

**Methods:**

Plasma samples and clinical parameters were obtained from 50 acute malaria cases (nine severe and 41 uncomplicated cases) from Thailand at three time points: day 0, day 7 and day 28. Gal-9 levels were determined by ELISA. A total of 38 species of cytokines and chemokines were measured using a BioPlex assay.

**Results:**

Gal-9 levels were higher at day 0 compared to day 7 and day 28 (P < 0.0001). Gal-9 levels were also higher in severe malaria (SM) cases compared to uncomplicated (UM) cases at day 0 and day 7 (923 vs 617 pg/mL; P = 0.03, and 659 vs 348 pg/mL; P = 0.02 respectively). Median Gal-9 levels were higher in patients with blood urea nitrogen to creatinine ratio (BUN/creatinine) ≥20 (mg/dL) than in patients with BUN/creatinine <20 (mg/dL) at day 0 (817.3 vs 576.2 pg/mL, P = 0.007). Gal-9 was inversely significantly correlated with chloride levels in both SM and UM cases (*r*_s_ = −0.73 and *r*_s_ = −0.46, respectively). In both UM and SM cases, Gal-9 was significantly associated with pro- and anti-inflammatory cytokines and chemokines such as TNF, IL-6, IFN-α2, IFN-γ, IL-1Ra and IL-10. These correlations were observed at day 0 but disappeared at day 28.

**Conclusions:**

Gal-9 is released during acute malaria, and reflects its severity. This elevation of Gal-9 in acute malaria infection raises the possibility of its role in termination of the immune response by binding to Tim-3, a receptor of Gal-9.

## Background

Malaria infection is still a big challenge for public health; around 214 million new cases of malaria worldwide occurred in 2015 (range 149–303 million) [1]. *Plamosdium falciparum* is responsible for the most severe cases of malaria and the greatest number of deaths. Detection and monitoring of severe cases are remaining issues for patient treatment [2].

Galectin-9 (Gal-9) is member of the galectin family of β-galactoside-binding animal lectins with a conserved carbohydrate recognition domain (CRD) [3]. Gal-9 was first described as an eosinophilic chemoattractant [4]. Gal-9 is presumed to bind a variety of molecules including glycoproteins and glycolipids of immune cells and pathogens [5]. These binding properties exert a variety of physiological and pathological functions such as cell differentiation, adhesion, aggregation, and cell death [6]. The elevation of plasma Gal-9 levels during acute human immunodeficiency virus (HIV) infection followed by a rapid decrease after anti-retroviral therapy was reported [7, 8]. Increase in plasma Gal-9 has also been seen during acute dengue virus infection [9]. Gal-9 inhibits production of several pro-inflammatory cytokines (TNF, IL-6, IL-1α) and enhances production of IL-10 [10]. These cytokines are believed to be involved in pathophysiology of malaria [11–13]. Recent findings of co-inhibitory or immune checkpoint receptors have been giving a novel insight into immune regulation. Because these receptors have a critical role in the maintenance of immune homeostasis: their expression on effector T cells ensures the proper contraction of effector T cells responses and their expression on regulatory T (Treg) cells guarantees the proper function of Treg cells to control effector T cells [14, 15].

It has been reported that PD1, a co-inhibitory receptor play a major role for inactivating a protective immunity in malaria infection in mice [16]. Gal-9 binds to T cell immunoglobulin and domain 3 (Tim-3) a co-inhibitory receptor, and that binding can ameliorate experimental autoimmune encephalomyelitis (EAE) by inducing cell death in Tim-3^+^ Th1 cells [17, 18]. Gal-9 also regulates activated CD8+ T cells [19], which increase during malaria [20]. Activation of CD8+ T cells these cells is considered as a risk factor of anemia in severe malarial [21]. These emerging regulatory activities of Gal-9 involving co-inhibitory receptors took us to investigate Gal-9 in malaria, though how Tim-3 functions to determine effector T cell responses is not yet well clarified [15]. Recently expression of Gal-9 and Tim-3 was demonstrated in lung, mediastinal lymph nodes and liver tissues in murine malaria model [22, 23]; however, the plasma levels of Gal-9 in human malaria patients has not been evaluated yet.

In this study, the plasma levels of Gal-9 in malaria patients were measured at three time points (days 0, 7 and 28) to examine its kinetics and association with cytokines, chemokines and clinical parameters.

## Methods

### Study subjects

Samples at three time points (day 0, day 7 and day 28) were obtained from 50 malaria patients, of which 41 were acute uncomplicated falciparum malaria (UM) and nine were severe falciparum malaria (SM) cases. The median age of patients enrolled was 23 (range 15–50) years old. There were 29 males and 21 females. Patient body temperatures were >38.5 °C upon admission. Diagnosis was performed by thick and thin blood smear in patients presenting malaria symptoms and malaria cases were defined by positive asexual forms of *P. falciparum*. Categorization of malaria cases was performed using WHO criteria [24]. Blood was collected in EDTA-containing tubes and centrifuged at 3000 rpm for 10 min. Obtained plasma was aliquoted and stored at −80 °C until use. On day 0, blood was collected and all patients were initiated on treatment with artemisinin-based combination therapy (ACT). Patients were followed up until day 28 according to the protocol.

### Clinical processing

At enrollment, clinical information was taken and entered in standardized forms. Clinical and bedside physical examinations were performed. Complete blood counts (CBC) in addition to kidney and liver function tests were examined.

### Biomarker measurement

Gal-9 concentration in plasma was measured using an ELISA (Galpharam Co. Ltd., Takamatsu, Japan) as previously described [9]. Microplates with 96 wells were coated with anti-human Gal-9 monoclonal antibody (9S2-3), blocked with 5 % fetal bovine serum in PBS, then incubated with the test sample (eightfold diluted plasma) for 1 h at RT with continuous shaking at 225 rpm. After several washing steps, Gal-9 remaining in the wells was recognized by a polyclonal anti-human Gal-9 antibody conjugated with biotin using EZ-Link Sulfo-NHS-Biotin reagent (Pierce). Quantification was performed by incubating wells with streptavidin-conjugated horseradish peroxidase (Invitrogen, Tokyo, Japan) and the colorimetric substrate tetramethylbenzidine (KPL, Gaitherburg, MD); then, the optical density was read with a microplate spectrophotometer (Bio-Rad).

Thirty-eight cytokine and chemokine species were measured using a commercially available kit (Milliplex Human Cytokine and Chemokine multiplex assay kit, Merck Millipore, Billerica, MA, USA) by Luminex methods as previously [9]. The assay was performed according to manufacturer’s instructions and the concentrations of cytokines/chemokines were calculated by comparing reads with a 5-parameter logistic standard curve using a Bioplex-200 instrument (Bio-Rad, Hercules, CA, USA).

### Statistical analysis

The distribution of data did not generally show a normal distribution by using the Kolmogorov–Smirnov test. Therefore, the data were expressed as the median and range. Friedman test, a non-parametric statistical test, was used to assess differences of Gal-9 and other biomarkers levels between different time points. The Mann–Whitney test, also a non-parametric test, was used to assess differences in biomarkers and clinical parameters between SM and UM cases. Correlation was assessed using Spearman’s rank correlation coefficient test. These statistical analyses were performed using GraphPad Prism 6 software (GraphPad Software, San Diego, CA, USA). A significant difference was assumed when *P* < 0.05.

## Results

### Clinical parameter profiles in uncomplicated and severe malaria cases

The median of haemoglobin were not significantly different between UM and SM cases at day 0 (Table 1). However, at day-7, the median haemoglobin level in SM cases was significantly lower compared to UM cases. Direct bilirubin median levels were higher in SM cases compared to UM cases at day 0 and day 7. Median platelet level was significantly lower at day 0 in SM compared to UM cases. The median levels of creatinine, BUN and AST level were significantly higher in SM cases compared to UM at day 0. Median levels of sodium and bicarbonate were lower in SM cases compared to UM at day 0 (Table 1).

**Table 1:** Clinical parameters of uncomplicated and severe malaria patients from day-0 to day-7

Clinical parameters^a^	Day 0		Day 7	
	UM	SM	UM	SM
Haemoglobin (g/dL)	11.9 (6–14.4)	11 (6.3–14)^b^ ns	10.3 (6.3–14.2)	8.6 (6.3–9.7)^c,^ **
Direct Bilirubin (mg/dL)	0.29 (0.06–2.6)	0.98 (0.3–5.7)^b,^ ***	0.12 (0.06–0.55)	0.235 (0.1–0.64)^c,^ *
Platelets (cell/μL)	85.5 (16–502)	27 (16–59)^b,^ **	249.5 (138–563)	226 (130–429)^c^ ns
Creatinine (mg/dL)	1 (0.6–1.6)	1.25 (1–6.4)^b,^ **	0.8 (0.5–1.3)	0.775 (0.6–1.15)^c^ ns
BUN (mg/dL)	19.5 (6–56.7)	44.5 (20.3–154.4)^b,^ ****	8.5 (4.5–16.5)	9 (5–12)^c^ ns
AST (U/L)	35 (18–230)	80 (40–295)^b,^ **	34.00 (12.00–97)	43.50 (20–295)^c^ ns
Sodium (mmol/L)	135 (118–147)	129.5 (118–144)^b,^ *	140 (136–146)	138 (134–144)^c^ ns
Bicarbonate (mmol/L)	22 (15–23)	20 (17–25)^b,^ *	22 (20–26)	21 (21–24)^c^ ns

Mann–Whitney non-parametric test was used to assess differences between parameters

*UM* uncomplicated malaria; *SM* severe malaria

**** P < 0.0001, *** P < 0.001, ** P < 0.01, and * P < 0.05

^a^Median and range

^b^Compared UM to SM on day 0

^c^UM to SM 7

### Kinetic changes in plasma levels of galectin-9 and description of its levels in uncomplicated and severe malaria cases

**Fig. 1:**
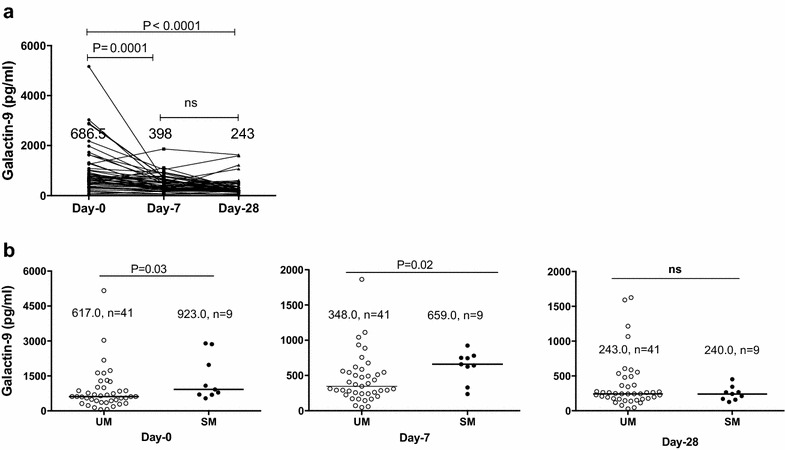
Plasma galectin-9 levels in patients with malaria. **a** Galectin-9 kinetics from day 0 to day 28. **b** Comparison between uncomplicated and severe cases, results are expressed as the median. *UM* uncomplicated malaria, *SM* severe malaria

Median plasma levels of Gal-9 were significantly elevated in malaria patients at day 0 (686 pg/mL) compared to day 7 (398 pg/mL) and day 28 (243 pg/ml). The median levels of Gal-9 on day 7 was found to be higher compared to day 28 but the difference was not significant (Fig. 1a). Median levels of Gal-9 were significantly higher in SM compared to UM at day 0 (923 pg/mL vs 617 pg/mL) and day 7 (659 pg/mL vs 348 pg/mL*).* At day 28, there was no significant difference in the Gal-9 levels between the two groups (Fig. 1b).

### Kinetic changes of other biomarkers

Among the 38 biomarkers measured, pro-inflammatory cytokines of IFN-α2, IL-1α, TNF and IL-6, Th1-related cytokine of IFN-γ, regulatory cytokines of IL-10 and IL-1Ra, growth cytokines of G-CSF, GM-CSF, EGF, FGF-2 and VEGF, and chemokines of IP-10, MIP-1α, MIP-β, MCP-1, IL-8, eotaxin, GRO, Fractalkine and MDC were detectable. All of them showed significant difference at day 0 compared to day 7 or day 28 except VEGF, MIP-1α, eotaxin and GRO (Table 2). In addition, the levels of MDC, EGF and sCD40L showed significant increase at day 28 compared to day 0 (Table 2).

**Table 2:** Kinetic changes of biomarkers from day-0 to day-28

Biomarkers	Day-0 (median)	Day-7 (median)	Day-28 (median)
Pro-inflammatory cytokines			
IFN-α2	19.95 (2.980–173.9)^a,^ ****	11.92 (2.980–79.51)^b^ ns	8.87 (2.980–70.26)^c,^ ****
IL-1α	25.32 (2.77–69.02)^a,^ **	10.28 (2.44–103.4)^b^ ns	9.63 (2.44–124.9)^c,^ **
TNF	36.54 (3.91–185.5)****	10.72 (3.83–74.45)^b^ ns	9.035 (3.89–74.45)^c,^ ****
IL-6	55.11 (4.240–1814)^a^ ns	7.57 (4.74–77.81)^b^ ns	11.51 (4.74–77.81)^c,^ **
Th1-related cytokines			
IFN-γ	28.6 (3.44–616)^a,^ ****	10.3 (3.14–185.2)^b^ ns	9.2 (3.44–150)^c,^ ****
Regulatory cytokines			
IL-10	549 (7.94–2321)^a,^ ****	72.66 (3.12–281.9)^b,^ *	20.54 (3.390–2576)^c,^ ****
IL-1Ra	109.8 (20.47–1897)^a,^ ****	31.85 (5.47–1358)^b^ ns	19.64 (3.68–595.4)^c,^ ****
sCD40L	851 (70.01–18,117)^a,^ ****	24,316 (96.21–297,336)^b^ ns	1762 (54.78–200,426)^c,^ ****
Growth cytokines			
G-CSF	65.85 (6.66–182)^a,^ ****	20.1 (5.38–197)^b^ ns	18.98 (3.93–132.9)^c,^ ****
GM-CSF	15.23 (4.420–180)^a,^ *	8.9 (3.810–95)^b^ ns	6.54 (3.090–85.38)^c,^ ****
EGF	13.72 (4.060–56.37)^a,^ *	23.6 (4.060–482.4) 8^b^ ns	42.17 (6.4–344)^c,^ ****
FGF-2	37.05 (13.04–507)^a^ ns	35.51 (8.12–289.2)^b,^ **	27.29 (4.59–171.8)^c,^ ****
VEGF	104.1 (23.5–2204)^a^ ns	99.74 (2.24–1754)^b^ ns	77.51 (18.07–3426)^c^ ns
TGF-α	4.69 (0.63–21.95)^a,^ *	4.02 (0.29–34.2)^b^ ns	3.18 (0.44–10.49)^c,^ *
Chemokines			
IP-10	3036 (155–8700)^a,^ ****	671.8 (28.54–1354)^b,^ * ns	449 (110.2–6002)^c,^ ****
MIP-1α	25.11 (3.750–85.07)^a^ ns	12.11 (3.780–216)^b^ ns	9.675 (3.47–144.7)^c^ ns
MIP-1β	50.8 (13.23–199.8)^a,^ ****	24.78 (4.950–172.5)^b^ ns	25.42 (4.26–187.2)^c,^ ****
MCP-1	591.1 (40.67–9148)^a,^ ****	228.2 (50.68–462.7)^b,^ ***	323.9 (52.06–1049)^c,^ *
IL-8	35.15 (3.49–227.8)^a,^ *	19.82 (4.05–604)^b^ ns	10.46 (3.65–564)^c,^ *
Eotaxin	161.3 (21.35–1840)^a^ ns	109.4 (6.54–264.5)^b^ ns	122 (27.25–327)^c^ ns
GRO	684.6 (184.5–1701)^a^ ns	806.3 (288.8–2962)^b^ ns	799.3 (238.8–1902)^c^ ns
Fractalkine	77.84 (12.51–546.4)^a,^ ****	25.36 (5.97–533.2)^b^ ns	25.79 (2–340)^c,^ ****
MDC	284.2 (29.29–1049)^a^ ns	317.8 (64.74–1595)^b,^ ****	502.4 (75.40–801.5)^c,^ ****

Results are shown in median levels with range

Friedman test was used to assess differences between days

**** P < 0.0001, *** P < 0.001, ** P < 0.01, and * P < 0.05

^a^Day 0 vs day 7

^b^Day 7 vs day 28

^c^Day 0 vs day 28

### Association of galectin-9 with various biomarkers

Numerous cytokines and chemokines were correlated with Gal-9 levels in malaria patients at day 0 (Table 3). At day 0, IFN-α2 was positively associated with Gal-9 only in SM cases (r_s_ = 0.82). TNF and IL-6 were positively correlated with Gal-9 in both UM (r_s_ = 0.57 and r_s_ = 0.36, respectively) and SM cases (r_s_ = 0.83 and r_s_ = 0.73, respectively). IFN-γ was positively associated with Gal-9 in both UM (r_s_ = 0.33) and SM cases (r_s_ = 0.75) (Table 3). IL-10 and IL-1Ra were positively associated with Gal-9 in both UM (r_s_ = 0.43 and r_s_ = 0.31, respectively) and SM cases (r_s_ = 0.82; and r_s_ = 0.83, respectively).

**Table 3:** Correlation of galectin-9 with cytokines and chemokines at day 0

Biomarkers	Galectin-9			
	UM		SM	
	r_s_	*P* value	r_s_	*P* value
IFN-α2	0.21	ns	0.82	0.01
TNF	0.57	0.0001	0.83	0.008
IL-6	0.36	0.03	0.73	0.03
IFN-γ	0.33	0.04	0.75	0.02
IP-10	0.4	0.009	0.65	ns
IL-8	0.44	0.004	0.88	0.0031
IL-10	0.43	0.004	0.82	0.01
IL-1Ra	0.31	0.05	0.83	0.008
G-CSF	0.35	0.03	0.9	0.002
MIP-1β	0.32	0.04	0.88	0.003
MCP-1	0.32	0.04	0.55	ns
Fractalkine	0.27	ns	0.72	0.04

Correlation was calculated using a non-parametric Spearman’s correlation test

*UM* uncomplicated malaria; *SM* severe malaria

In addition, Gal-9 was also correlated significantly with G-CFS and MIP-1β in both UM (r_s_ = 0.35 and r_s_ = 0.32, respectively) and SM cases (r_s_ = 0.9 and r_s_ = 0.88, respectively). Gal-9 also correlated with MCP-1 in UM group (r_s_ = 0.32) and Fractalkine in SM cases (r_s_ = 0.72) (Table 3).

### Association of plasma galectin-9 with clinical parameters of malaria infection

**Fig. 2:**
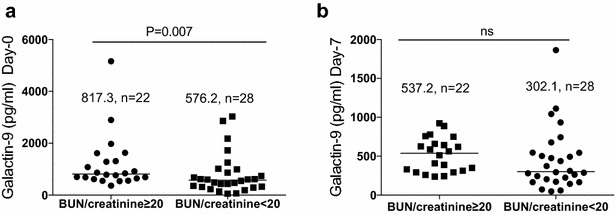
Comparison of galectin-9 levels on the basis of BUN/creatinine ratio **a** distribution of Gal-9 on day 0 according  BUN/creatinine. **b** distribution of Gal-9 on day 7 according to BUN/creantinine on day-0. Mann–Whitney non-parametric test was used to assess significances. Results are expressed as the median

Gal-9 levels on day 0 were negatively correlated with albumin (r_s_ = −0.69), and were positively correlated with creatinine (r_s_ = 0.44) and BUN (r_s_ = 0.45) in the UM cases. The lack of correlation between these kidney parameters and Gal-9 in SM cases might be due to the small number of cases in that group (nine patients). Among the electrolytes tested, sodium levels were negatively associated with Gal-9 levels (r_s_ = −0.48) also only in the UM cases. Chloride levels were negatively associated with Gal-9 in both UM (r_s_ = −0.46) and SM cases (r_s_ = −0.73) (Table 4). Furthermore, the Gal-9 median level in patients with BUN/creatinine ≥20 (mg/dL) (pre-renal azotaemia) was significantly higher than those with BUN/creatinine <20 (mg/dL) at day 0 (817.3 vs 576.2 pg/mL) but not at day 7 (Fig. 2).

**Table 4:** Correlation between galectin-9 and clinical parameters

Clinical parameter	Galectin-9			
	UM		SM	
	*r*_*s*_	*P* value	*r*_*s*_	*P* value
Albumine	−0.69	<0.0001	−0.29	ns
Creatinine	0.44	0.005	0.32	ns
BUN	0.45	0.004	0.43	ns
AST	0.46	0.003	0.55	ns
Sodium	−0.48	0.005	−0.25	ns
Chloride	−0.46	0.006	−0.73	0.04

Correlation was calculated using a non-parametric Spearman’s correlation test. *r*_*s*_ value represents correlation coefficient. *P* value represents significance level

*UM* uncomplicated malaria; *SM* severe malaria

## Discussion

This study revealed the presence of Gal-9 in plasma in malaria patients and showed that plasma levels of Gal-9 accurately reflect the status of inflammation and recovery during malaria infection for the first time though the tissue expression of Gal-9 and Tim-3 was demonstrated in murine malaria model [21, 22]. The elevation of Gal-9 was reported in other acute and non-acute infectious diseases such as dengue virus infection (median level was 1525 pg/mL), leptospirosis (616 pg/mL) [9], acute HIV infection (~2300 pg/mL) [8] and active tuberculosis (200 pg/mL) [25]. Detailed analysis with clinical parameters in dengue virus infections showed its association with the clinical severity. Gal-9 exerts its pivotal immunomodulatory effects by inducing apoptosis or suppressing effector functions via engagement with its receptor, Tim-3 a co-inhibitory receptor, though how Tim-3 functions to determine effector responses have not been clarified, yet [14, 15]. The versatile activities of Gal-9 was attributed by dual activities of the molecule, anti-microbial as well as anti-inflammatory activities [26].

It has been suggested that Gal-9 production might be stimulated by the response of mounting Th1 cells that occurs during *P. falciparum* infection, and produced Gal-9 was assumed to inhibit their activation [10, 14]. This may explain the correlation seen in this study between Gal-9 and inflammatory cytokines (IFN-γ, TNF, IFN-α and IL-6) and chemokines (MIP-1β, MCP-1 and Fractalkine) at day 0. It was proposed that the levels of TNF and IL-6 are indicators of malaria severity [11]. This association of these molecules with Gal-9 in both UM and SM cases supports the idea of Gal-9 as a severity marker in malaria infection. In fact, Gal-9 levels correlated positively with kidney parameters and inversely with electrolytes.

The correlation of Gal-9 with IL-10 could be related to the effect of Gal-9 on differentiation of naïve T cells to Gal-9^+^ ThGal and Treg, which express high levels of IL-10 mRNA [27]. Though both Gal-9 and IL-10 were proposed to have anti-inflammatory properties, it is known that IL-10 production is impaired in SM compared with UM as reported elsewhere [28]. Another anti-inflammatory cytokine IL-1Ra levels correlated with Gal-9 both in UM and SM cases and IL-1Ra was found to be correlated with parasitemia [29]. In malaria, a variety immune cells are known to be activated such as CD8+ T, CD4+ T and NK cells [30–32], and their function can be regulated or impaired by Gal-9 in vitro [19, 33]. However, it is difficult to demonstrate if the released Gal-9 could constrain immune response through the Gal-9/Tim-3 pathway [19]. Gal-9 shares the receptor Tim-3 with high mobility box group-1 (HMBG-1), an inflammation mediator [14, 34]. HMGB-1 elevation was reported an as informative prognostic marker for disease severity in human severe malaria [35]. In contrast to the apoptotic effect of Gal-9 on T cells [14], HMGB-1 has been described as having proliferation effects by binding through HMGB-1/Tim-3 and HMGB-1/RAGE on T cells [36, 37]. The differential roles of these two ligands (HMGB-1 and Gal-9) in controlling the immune response against parasites should be clarified in future studies.

Beside immune modulatory activities, Gal-9 is released from infected or damaged cells, and could act as danger signal molecules [9], by initiating inflammation as reported in murine respiratory tularaemia [38]. Moreover the Tim-3-Gal-9 expression was found to be elevated in acute lung injury in a murine malarial model [22].

The released Gal-9 can also bind to galactosides of pathogens, pathogen-associated molecular pattern (PAMP), as pathogen recognition receptors (PRRs), as shown recognition of the *Leishmania major* Poly-β-galactosyl epitope by Gal-9 [39]. Future Investigation on the biological roles of Gal-9 in malaria infection would be necessary.

## Conclusions

Taken together, this study shows an increase of plasma Gal-9 levels during malaria infection, which is capable of identifying severe cases, and is tracking the inflammation process. Therefore, it may be used as a novel biomarker of malaria infection.

## Abbreviations

UM: uncomplicated malaria
SM: severe malaria
Gal-9: galectin-9
Tim-3: T cell immunoglobulin and domain 3
HMGB-1: high-mobility group box 1
TNF: tumour necrosis factor
IFN: interferon
IP-10: interferon inducible protein 10
IL: interleukin
IL-1ra: interleukin-1 receptor antagonist
GM-CSF: granulocyte monocyte colony stimulating factor
G-CSF: granulocyte colony stimulating factor
MIP: macrophage inflammatory protein
MDC: macrophage-derived chemokine
MCP: monocyte chemoattractant protein
TGF: transforming growth factor
EGF: epidermal growth factor
sCD40L: soluble cluster of differentiation 40 ligand
BUN: blood urea nitrogen

## References

[1] WHO (2015). World malaria report 2015.

[2] WHO (2012). Management of severe malaria: a practical handbook.

[3] Su EW, Bi S, Kane LP (2011). Galectin-9 regulates T helper cell function independently of Tim-3. Glycobiology.

[4] Matsumoto R, Matsumoto H, Seki M, Hata M, Asano Y, Kanegasaki S (1998). Human ecalectin, a variant of human galectin-9, is a novel eosinophil chemoattractant produced by T lymphocytes. J Biol Chem.

[5] Vasta GR (2012). Galectins as pattern recognition receptors: structure, function, and evolution. Adv Exp Med Biol.

[6] Niwa H, Satoh T, Matsushima Y, Hosoya K, Saeki K, Niki T (2009). Stable form of galectin-9, a Tim-3 ligand, inhibits contact hypersensitivity and psoriatic reactions: a potent therapeutic tool for Th1- and/or Th17-mediated skin inflammation. Clin Immunol.

[7] Saitoh H, Ashino Y, Chagan-Yasutan H, Niki T, Hirashima M, Hattori T (2012). Rapid decrease of plasma galectin-9 levels in patients with acute HIV infection after therapy. Tohoku J Exp Med.

[8] Chagan-Yasutan H, Saitoh H, Ashino Y, Arikawa T, Hirashima M, Li S (2009). Persistent elevation of plasma osteopontin levels in HIV patients despite highly active antiretroviral therapy. Tohoku J Exp Med.

[9] Chagan-Yasutan H, Ndhlovu LC, Lacuesta TL, Kubo T, Leano PS, Niki T (2013). Galectin-9 plasma levels reflect adverse hematological and immunological features in acute dengue virus infection. J Clin Virol.

[10] Seki M, Oomizu S, Sakata KM, Sakata A, Arikawa T, Watanabe K (2008). Galectin-9 suppresses the generation of Th17, promotes the induction of regulatory T cells, and regulates experimental autoimmune arthritis. Clin Immunol.

[11] Kern P, Hemmer CJ, Van Damme J, Gruss HJ, Dietrich M (1989). Elevated tumor necrosis factor alpha and interleukin-6 serum levels as markers for complicated *Plasmodium falciparum* malaria. Am J Med.

[12] Kwiatkowski D, Hill AV, Sambou I, Twumasi P, Castracane J, Manogue KR (1990). TNF concentration in fatal cerebral, non-fatal cerebral, and uncomplicated *Plasmodium falciparum* malaria. Lancet.

[13] Wilson NO, Bythwood T, Solomon W, Jolly P, Yatich N, Jiang Y (2010). Elevated levels of IL-10 and G-CSF associated with asymptomatic malaria in pregnant women. Infect Dis Obstet Gynecol.

[14] Zhu C, Anderson AC, Schubart A, Xiong H, Imitola J, Khoury SJ (2005). The Tim-3 ligand galectin-9 negatively regulates T helper type 1 immunity. Nat Immunol.

[15] Anderson AC, Joller N, Kuchroo VK (2016). Lag-3, Tim-3, and TIGIT: Co-inhibitory receptors with specialized functions in immune regulation. Immunity.

[16] Horne-Debets JM, Karunarathne DS, Faleiro RJ, Poh CM, Renia L, Wykes MN (2016). Mice lacking programmed cell death-1 show a role for CD8 (+) T cells in long-term immunity against blood-stage malaria. Sci Rep.

[17] Dardalhon V, Anderson AC, Karman J, Apetoh L, Chandwaskar R, Lee DH (2010). Tim-3/galectin-9 pathway: regulation of Th1 immunity through promotion of CD11b + Ly-6G + myeloid cells. J Immunol.

[18] Monney L, Sabatos CA, Gaglia JL, Ryu A, Waldner H, Chernova T (2002). Th1-specific cell surface protein Tim-3 regulates macrophage activation and severity of an autoimmune disease. Nature.

[19] Sehrawat S, Reddy PB, Rajasagi N, Suryawanshi A, Hirashima M, Rouse BT (2010). Galectin-9/TIM-3 interaction regulates virus-specific primary and memory CD8 T cell response. PLoS Pathog.

[20] Hojo-Souza NS, Pereira DB, Passos LS, Gazzinelli-Guimaraes PH, Cardoso MS, Tada MS (2015). Phenotypic profiling of CD8 (+) T cells during *Plasmodium vivax* blood-stage infection. BMC Infect Dis.

[21] Safeukui I, Gomez ND, Adelani AA, Burte F, Afolabi NK, Akondy R (2015). Malaria induces anemia through CD8+ T cell-dependent parasite clearance and erythrocyte removal in the spleen. MBio.

[22] Liu J, Xiao S, Huang S, Pei F, Lu F (2016). Upregulated Tim-3/galectin-9 expressions in acute lung injury in a murine malarial model. Parasitol Res.

[23] Xiao S, Liu J, Huang S, Lu F (2016). Increased Gal-9 and Tim-3 expressions during liver damage in a murine malarial model. Parasitol Res.

[24] WHO (2015). Guidelines for the treatment of malaria.

[25] Shiratori B, Leano S, Nakajima C, Chagan-Yasutan H, Niki T, Ashino Y (2014). Elevated OPN, IP-10, and neutrophilia in loop-mediated isothermal amplification confirmed tuberculosis patients. Mediators Inflamm.

[26] Merani S, Chen W, Elahi S (2015). The bitter side of sweet: the role of Galectin-9 in immunopathogenesis of viral infections. Rev Med Virol.

[27] Oomizu S, Arikawa T, Niki T, Kadowaki T, Ueno M, Nishi N (2012). Cell surface galectin-9 expressing Th cells regulate Th17 and Foxp3+ Treg development by galectin-9 secretion. PLoS One.

[28] Boeuf PS, Loizon S, Awandare GA, Tetteh JK, Addae MM, Adjei GO (2012). Insights into deregulated TNF and IL-10 production in malaria: implications for understanding severe malarial anaemia. Malar J.

[29] Cox-Singh J, Singh B, Daneshvar C, Planche T, Parker-Williams J, Krishna S (2011). Anti-inflammatory cytokines predominate in acute human *Plasmodium knowlesi* infections. PLoS One.

[30] Miyakoda M, Kimura D, Yuda M, Chinzei Y, Shibata Y, Honma K (2008). Malaria-specific and nonspecific activation of CD8+ T cells during blood stage of *Plasmodium berghei* infection. J Immunol.

[31] Perez-Mazliah D, Langhorne J (2014). CD4 T-cell subsets in malaria: TH1/TH2 revisited. Front Immunol.

[32] Chen Q, Amaladoss A, Ye W, Liu M, Dummler S, Kong F (2014). Human natural killer cells control *Plasmodium falciparum* infection by eliminating infected red blood cells. Proc Natl Acad Sci USA.

[33] Folgiero V, Cifaldi L, Li Pira G, Goffredo BM, Vinti L, Locatelli F (2015). TIM-3/Gal-9 interaction induces IFNgamma-dependent IDO1 expression in acute myeloid leukemia blast cells. J Hematol Oncol.

[34] Chiba S, Baghdadi M, Akiba H, Yoshiyama H, Kinoshita I, Dosaka-Akita H (2012). Tumor-infiltrating DCs suppress nucleic acid-mediated innate immune responses through interactions between the receptor TIM-3 and the alarmin HMGB1. Nat Immunol.

[35] Higgins SJ, Xing K, Kim H, Kain DC, Wang F, Dhabangi A (2013). Systemic release of high mobility group box 1 (HMGB1) protein is associated with severe and fatal *Plasmodium falciparum* malaria. Malar J.

[36] Dumitriu IE, Baruah P, Valentinis B, Voll RE, Herrmann M, Nawroth PP (2005). Release of high mobility group box 1 by dendritic cells controls T cell activation via the receptor for advanced glycation end products. J Immunol.

[37] Chen Y, Akirav EM, Chen W, Henegariu O, Moser B, Desai D (2008). RAGE ligation affects T cell activation and controls T cell differentiation. J Immunol.

[38] Steichen AL, Simonson TJ, Salmon SL, Metzger DW, Mishra BB, Sharma J (2015). Alarmin function of galectin-9 in murine respiratory tularemia. PLoS One.

[39] Pelletier I, Hashidate T, Urashima T, Nishi N, Nakamura T, Futai M (2003). Specific recognition of *Leishmania major* poly-beta-galactosyl epitopes by galectin-9: possible implication of galectin-9 in interaction between L. major and host cells. J Biol Chem.

